# Snapshots of the second-step self-splicing of *Tetrahymena* ribozyme revealed by cryo-EM

**DOI:** 10.1038/s41467-023-36724-5

**Published:** 2023-03-16

**Authors:** Shanshan Li, Michael Z. Palo, Xiaojing Zhang, Grigore Pintilie, Kaiming Zhang

**Affiliations:** 1 Department of Urology, The First Affiliated Hospital of USTC, MOE Key Laboratory for Cellular Dynamics, Hefei National Research Center for Interdisciplinary Sciences at the Microscale, Division of Life Sciences and Medicine, University of Science and Technology of China, Hefei, 230001 China; 2 Department of Biochemistry, Stanford University, Stanford, CA 94305 USA; 3 Department of Bioengineering, Stanford University, Stanford, CA 94305 USA

**Keywords:** Ribozymes, Cryoelectron microscopy, RNA

## Abstract

Group I introns are catalytic RNAs that coordinate two consecutive transesterification reactions for self-splicing. To understand how the group I intron promotes catalysis and coordinates self-splicing reactions, we determine the structures of L-16 *Tetrahymena* ribozyme in complex with a 5′-splice site analog product and a 3′-splice site analog substrate using cryo-EM. We solve six conformations from a single specimen, corresponding to different splicing intermediates after the first ester-transfer reaction. The structures reveal dynamics during self-splicing, including large conformational changes of the internal guide sequence and the J5/4 junction as well as subtle rearrangements of active-site metals and the hydrogen bond formed between the 2′-OH group of A261 and the N2 group of guanosine substrate. These results help complete a detailed structural and mechanistic view of this paradigmatic group I intron undergoing the second step of self-splicing.

## Abstract (web summary)

The *Tetrahymena* ribozyme changes conformations to perform its self-splicing function. Here, the authors capture six structural snapshots of ribozyme during the second step of self-splicing from a single specimen, revealing the structural basis of how it promotes and coordinates self-splicing reactions.

## Introduction

Ribozymes are RNA molecules that catalyze specific biochemical reactions, such as RNA splicing, mRNA translation, and pre-tRNA processing. Group I introns catalyze their own excision from RNA precursors in many organisms through two sequential transesterification reactions^1^. The *Tetrahymena thermophila* group I self-splicing intron was the first ribozyme to be discovered^2^. Since its discovery in the 1980s, obtaining an atomic structure of the full-length molecule has been a challenge. Major milestones in understanding the structure of *Tetrahymena* ribozyme include the determination of a 2.8-Å crystal structure of the P4–P6 domain (helices P4, P5, and P6), which revealed that a sharp bend and long-range interactions allow these helices to form closely packed structures^3^. The 5.0-Å crystal structure for P3-P9 (helices P3, P7, P8, and P9) of *Tetrahymena* ribozyme suggested a preorganized active site for catalysis even in the absence of RNA substrates^4^. In addition, the crystal structure for P3–P9 at 3.8-Å further explicated how the active site places the metal ion (e.g. Mg^2+^) and nucleophilic guanosine for catalysis before substrate binding^5^. Furthermore, three-dimensional (3D) models of the whole architecture of the *Tetrahymena* ribozyme were first predicted based on sequence comparative analysis^6^ with some refinements proposed later for the P2.1–P3–P8 junction that folds as a right angle (RA) module^7^. The recent cryogenic electron microscopy (cryo-EM) structures of full-length *Tetrahymena* ribozyme determined at 6.8-Å^8^ and 3.1-Å^9^ mostly agree with previous structures but also reveal previously uncharacterized tertiary interactions^9^, demonstrating that cryo-EM single-particle analysis can be a powerful method for the determination of folded RNA structures at increasingly higher resolution.

Aside from the fact that *Tetrahymena* ribozyme folds into compact 3D structures, it promotes conformational changes among different functional states to facilitate the acceleration of chemical steps involved in self-splicing^10–13^. To date, extensive biochemical and genetic studies have elucidated the underlying catalytic mechanisms of the *Tetrahymena* ribozyme, but little is known about the detailed rearrangements in the 3D structure required for its catalytic functions. The high-resolution structures of *Tetrahymena* ribozyme in complex with its RNA substrates will be beneficial for understanding the structural basis of substrate recognition and uncovering the conformational changes that may occur after substrate binding. Given the unique advantage of cryo-EM single-particle analysis to resolve samples with high heterogeneity or flexibility^14,15^ and the fact that previous studies of ribozyme focus on a single holo state^9,16^, we aim to capture multiple structure snapshots of *Tetrahymena* ribozyme during the self-splicing process.

In this study, we solved the cryo-EM structures of the full-length *Tetrahymena* L-16 ScaI ribozyme in different states of self-splicing from a single specimen. Six conformations at 2.68 Å, 2.35 Å, 2.62 Å, 2.65 Å, 2.97 Å, and 3.41 Å resolution, respectively, were characterized. These resolutions enable us to determine atomically detailed structures of numerous states of the *Tetrahymena* ribozyme, revealing the structural basis of how it promotes catalysis and coordinates the self-splicing reactions.

## Results

### Single-particle cryo-EM analysis of *Tetrahymena* L-16 ScaI ribozyme

A shortened construct known as the L-21 ScaI ribozyme is the most commonly studied form of the *Tetrahymena* ribozyme. Although its active site is preserved, the L-21 ScaI ribozyme lacks a 5′-extension of the internal guide sequence (IGS), which forms dynamic helices involved in substrate recognition during self-splicing, including the P1 extension at the 5′-splice site in the first step of splicing and P10 at the 3′-splice site in the second step of splicing. Here, to better dissect the structural rearrangements in the self-splicing process, we used an IGS-extended ribozyme, the L-16 ScaI ribozyme that has the ability to form these dynamic helices^17^. Furthermore, given the capability of cryo-EM single-particle analysis to resolve heterogeneous structures, we added both the 5′-splice site analog product (S1; 5′cccucu3′) and 3′-splice site analog substrate (S2; 5′UCG*uaacc3′) to the L-16 ScaI ribozyme; in S2, the scissile phosphodiester bond between the guanosine (G) residue that occupies the G-binding site^18^ and the uracil (u) residue was substituted with a phosphorothioate bond (*) to slow down the rate, but not the extent, of the cleavage reaction^19,20^, which has been widely used to study RNA-directed catalysis, especially for a number of ribozymes^21–25^. These improvements allow us to capture different conformations from a single specimen during the second step of *Tetrahymena* intron splicing.

**Fig. 1:**
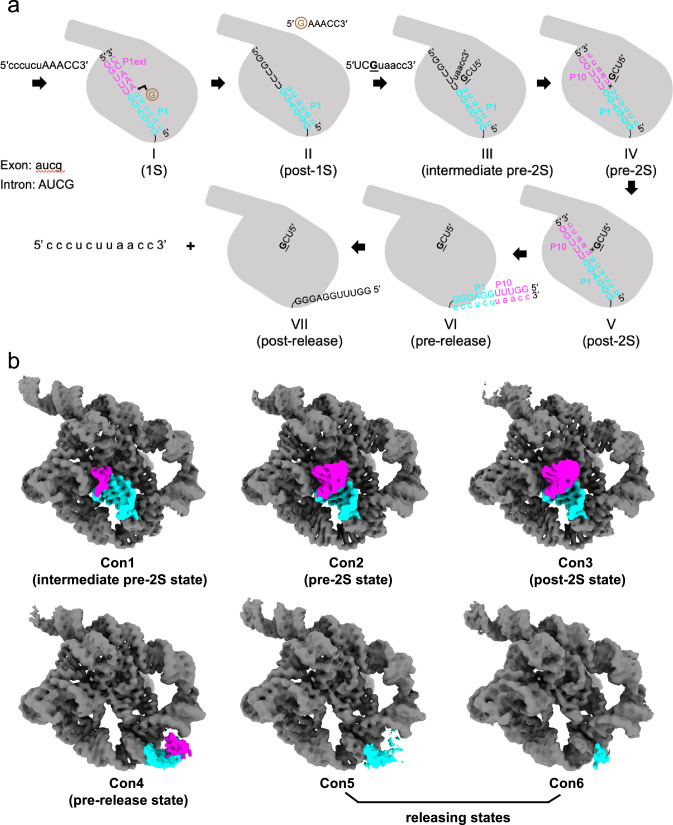
Snapshots of the self-splicing of *Tetrahymena* ribozyme revealed by cryo-EM. **a** Cartoon model of the self-splicing reaction of L-16 ScaI ribozyme. In the first transesterification reaction (I to II), an exogenous G (shown in brown) attacks the phosphodiester bond at the 5′-splice site in P1, leading to a free 3′-OH group at the upstream exon and the exogenous G being attached to the 5′-end of the intron. Then the 3′-splice site substrate G (shown in G) replaces the exogenous G in the G-binding site (III), and the 3′-exon is aligned in P10 for ligation (IV). In the second transesterification reaction (IV to V), the 3′-OH group of the upstream exon attacks the phosphodiester bond at the 3′-splice site in P10, resulting in the ligation of exons (V), followed by big conformational changes in the P1-P10 duplex (VI–VII) to release the intron (VII). Exon sequences are shown in lowercase and intron sequences are shown in UPPERCASE. 1S and 2S refer to the first and second steps of splicing, respectively. **b** Different self-splicing states (Con1-6) of *Tetrahymena* ribozyme resolved by cryo-EM.

To verify the catalytic ability of ribozyme, we carried out in vitro splicing assays and found that the product accumulates over time (Supplementary Fig. 1a). Then, the reaction time point of 5 h was selected to collect a large dataset including 25,306 movie stacks to obtain sufficient ribozyme particles, enabling us to classify different splicing states. During image processing, after Ab-initio 3D reconstruction and refinement in CryoSPARC, two discernible conformations with a noticeable difference in the 5′ end of the intron were derived (Supplementary Fig. 1b, c and Supplementary Fig. 2). Further 3D classification resolved six different conformations, occupying 5.1%, 57.7%, 4.9%, 18.4%, 10.0%, and 3.9% of total particles, respectively. These maps were further refined to 2.68 Å, 2.35 Å, 2.62 Å, 2.65 Å, 2.97 Å, and 3.41 Å, respectively (Supplementary Figs. 2–4). In three of these maps, the resolutions at the 5′ end of the intron are sufficient to show the atomic details (Con1-3 in Fig. 1 and Supplementary Fig. 4), allowing us to understand how conformational changes of *Tetrahymena* ribozyme facilitate the self-splicing process. Notably, the remaining maps show an undocked 5′-end with lower resolution (Con4-6 in Fig. 1 and Supplementary Fig. 4), indicating they are the apo or release state during the self-splicing reactions.

We then applied DRRAFTER^26^ to build the initial models for these maps, followed by optimization with Coot^27^ and Phenix^28^. Final models were evaluated using MolProbity^29^ (Supplementary Table 1) and Q-scores^30^ (Supplementary Fig. 5). Hereafter, to simplify the latter description, we referred to the six structures as Con1 (intermediate pre-2S state), Con2 (pre-2S state), Con3 (post-2S state), Con4 (pre-release state), as well as Con5 and Con6 (releasing states) according to the self-splicing process that they may represent (Fig. 1). Maps with a lower resolution at the 5′-end appeared to be complexes containing ligated substrates captured during product release.

### Partial P10 formation in docked *Tetrahymena* ribozyme

**Fig. 2:**
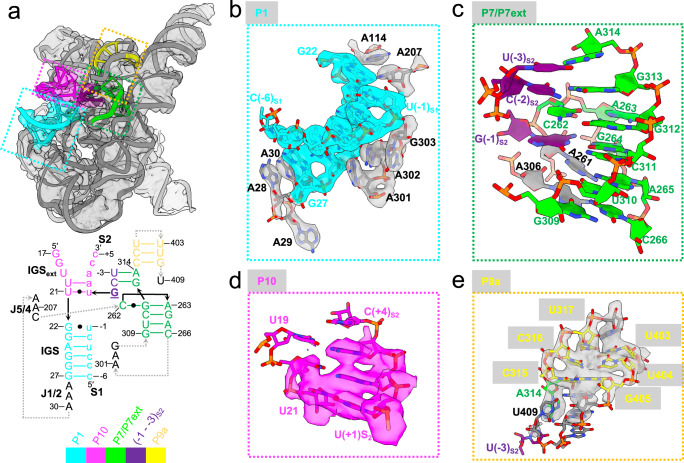
Structural features of *Tetrahymena* L-16 holoenzyme in Con1. **a** Cryo-EM map and model. The P1 helix, partial P10 helix, P7/P7ext, and P9a are highlighted, with their secondary structures shown below. Exon sequences are shown in lowercase and intron sequences are shown in UPPERCASE. **b** The intron’s IGS (G22-G27) interacts with S1 (u[−1]-c[−6]) to form the 6-bp P1 helix, which is stabilized by A301, A302, and G303 in J8/7. The U•G wobble is recognized by packed A207 and A114 in J4/5. **c** The G-binding site is composed of four layers of base triples (A263-C262-G312, G[−1]_S2_-G264-C311, A261-A265-U310, A306-C266-G309). The 2-bp P7 extension (C[−2]_S2_-G313 and U[−3]_S2_-A314) is stacked upon the A263-C262-G312 base triple. **d** Partial formation of the P10 helix. **e** The 3-bp P9a stem with the terminal U409 in coordination with A314 and U(−3)_S2_.

The G-binding site of *Tetrahymena* ribozyme is located in P7, a six-base pair helix with a single-base bulge (A263)^31^. In Con1 (Figs. 1 and 2a, Supplementary Fig. 6, and Supplementary Movie 1), the 5′-splice site analog product S1 forms six base pairs with the IGS at the 5′-end of the intron to form the helix P1, docking into the G-binding site. There is a sharp bend (~180°) between the nucleotides A28 and A29 in J1/2. A30 is positioned between G27-A28 and stacked on both sides (Fig. 2b). A301 and A302 from J8/7 continuously stack and make A-minor interactions with base pairs in P1; G303 also interacts with another base pair in P1 (Fig. 2b). Such tertiary interactions are critical for positioning the P1 helix. The last base pair of P1 is the U•G wobble, which is critical for specifying the 5′-splice site^32^. The U•G wobble was also proposed to promote the replacement of the P1 extension with P10 after the first transesterification reaction^17^.

In the G-binding site, G(−1)_S2_ forms a base triple with the G264-C311 base pair in P7 (Fig. 2c), consistent with the guanosine-to-G·C base triple postulated in a previous study^31^. Stacked upon this is another base triple formed by the bulged A263 and the C262-G312 base-pair. Underneath G(−1)_S2_, A261 located in J6/7 interacts with the A265-U310 base pair to make a third base triple in the G-binding site, followed by the fourth base triple A306-C266-G309 (Fig. 2c). This base triple sandwich was also formed in *Tetrahymena* ribozyme prior to RNA substrate binding^5,9^, indicating that the G-binding site is structurally well organized regardless of the presence of its substrate.

The P9.0 duplex is formed between the residues directly located at 5′ of the 3′-terminal G of the intron and single-stranded residues of the intron adjacent to the G-binding site^17^. It has been suggested to aid in the 3′-splice site recognition^33,34^. In our structure, 5′UCG of the S2 mimics the intron’s 3′-terminus and interacts with G313 and A314 in P7 to form a 2-bp P9.0 duplex-like stem, P7 extension (Fig. 2c). It likely promotes substrate binding by anchoring the strand close to the G-binding site and stacking upon the A263-C262-G312 base triple. This hypothesis is supported by the fact that the P7 extension (P7ext) formation precedes P10 formation, as demonstrated by the partial binding of S2 to IGSext (Fig. 2d). Besides, the high-resolution cryo-EM map allows us to confidently build the 3-bp P9a stem (Fig. 2e), which cannot be unambiguously resolved in our previous structures^9^. The coordination of the terminal U409 with U(−3)_S2_ further supports the structural rearrangement of the intron’s 3′-end for docking of the terminal G (ωG) into the active site.

### Pre- and post-catalysis states of *Tetrahymena* ribozyme

**Fig. 3:**
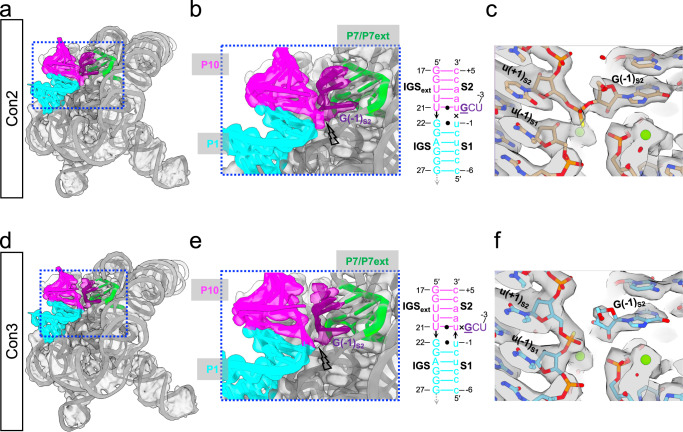
Structural features of *Tetrahymena* ribozyme in Con2 and Con3. **a**, **d** Cryo-EM map and model of Con2 (**a**) and Con3 (**d**). **b**, **e** Zoom-in views of P1 and P10 helices of Con2 (**b**) and Con3 (**e**). Their secondary structures are shown, with exon sequences shown in lowercase and intron sequences shown in UPPERCASE. The lightning symbol indicates the position of the cleaved bond. **c**, **f** Comparison of the density for the scissile phosphate between Con2 (**c**) and Con3 (**f**).

The phosphorothioate-substituted substrate, S2, allows us to capture the conformation poised for the second transesterification reaction (Con2), which is the major state that occupies 57.7% of total particles and the only state captured by our previous study^9^. In Con2 (Figs. 1 and 3a and Supplementary Movie 1), nucleotides +1 - +5 of the S2 make five base pairs with the IGSext to form the whole P10 helix. Both P1 and P10 helices dock into the active site, placing the 3′-splice site near the G-binding site (Fig. 3b, c and Supplementary Fig. 6), ready for the second step of splicing.

Although S2 has a phosphorothioate substitution at the scissile phosphate to slow down the reaction, it is a racemic mixture of both stereoisomers of the phosphorothioate, one of which slows down the reaction rate by a factor of >10,000 and the other by about 14^19^, therefore it is very likely that a fraction of complexes containing the latter stereoisomer has catalyzed the chemical step, leading to the *Tetrahymena* ribozyme Con3 (Fig. 1 and Fig. 3d and Supplementary Movie 1). In this conformation, the densities for S2-P7 interactions remain resolved, and the densities for the P7ext base pairs also appear at a lower threshold (Fig. 3e and Supplementary Fig. 6 and S7a). Strikingly different from Con2, in Con3 there is a clear break in the cryo-EM density between G(−1)_S2_ and u(+1)_S2_ and continuous density between u(−1)_S1_ and u(+1)_S2_, where a new phosphodiester bond should be formed (Fig. 3b, c, e, f). This supports the conclusion that Con3 has accomplished the second transesterification reaction, which leads to the exon ligation without major conformational changes compared to Con2. Notably, some extra density appears at the end of P10 at a lower threshold in Con3 (Supplementary Fig. 7b), which may be due to the flexibility of the P10 helix, favoring the detachment of the duplex from the active site. The P10 flexibility could be caused by the lack of tertiary contacts holding the P10 helix to the ribozyme active site after its breakage with 5′UCG_S2_.

### Undocked forms of *Tetrahymena* ribozyme

**Fig. 4:**
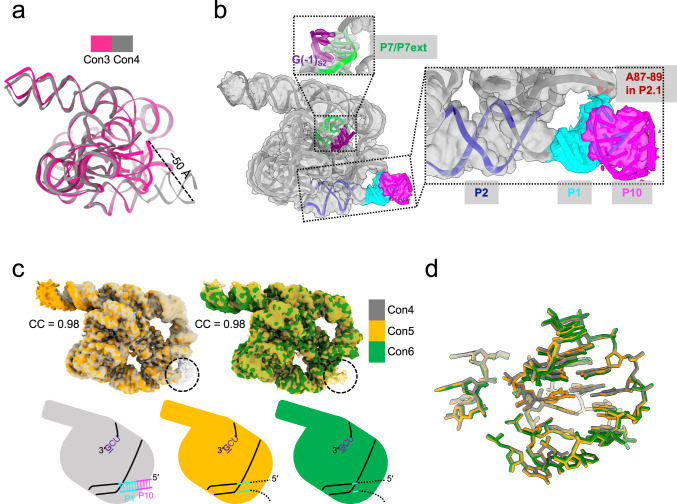
Structural features of *Tetrahymena* ribozyme in Con4-6. **a** The P1-P10 duplex undergoes a large conformational change in Con4 compared with Con3. **b** P1-P10 is coaxially stacked with P2 and contacts with P2.1, while the P7 extension remains unchanged. **c** Superpositions of Con4 and 5, as well as Con5 and 6 cryo-EM maps, reveal an unpairing process. Black dotted circles indicate undocked helical 5′-ends, dash lines refer to hydrogen bonds, and black dotted dash lines indicate unpaired nucleotides that are not resolved by cryo-EM. **d** Comparison of the catalytic core among Con4-6 models. Different conformations are shown using the same coloring scheme as in **c**.

Con4 shows an unbent 5′-end, indicating that the P1-P10 duplex has undergone substantial conformational changes compared with other conformations (Figs. 1 and 4a, Supplementary Fig. 6, and Supplementary Movie 1). The cryo-EM density of 5′UCG of the S2 remains resolved without density for the phosphorothioate (Fig. 4b), therefore we expect this conformation to be a post-reaction release state rather than a pre-docking state. It could be that following the ligation reaction, the P1-P10 duplex is released from the active site before 5′UCG. Notably, P1-P10 seems to be coaxially stacked with P2 and in contact with A87, A88, and A89 in P2.1 (Fig. 4b), which means that this duplex is detached from the active site and reaches the substrate-free site^35^ through a nearly 50-Å distance (Fig. 4a). The lower resolution at the 5′-end is likely caused by the inherent flexibility of the P1-P10 duplex due to the lack of stabilization from other tertiary contacts.

At the end of the ribozyme’s self-splicing process, the spliced product must be released. We captured two additional states (Con5-6) that may represent a substrate release process. There are high cross-correlation coefficients among Con4-6 maps (CC = 0.98) (Fig. 4c). At the nucleotide level, the major difference lies in the 5′-end of the intron, with negligible changes in the catalytic core (Fig. 4c, d). In Con5, only the first few base pairs of the P1-P10 duplex can be observed, which is even worse in Con6 (Fig. 4c and Supplementary Movie 1), indicating a possibility that an unpairing process has been ongoing in the P1-P10 duplex. The fact that undocked P1-P10 is stacked in continuity of P2 after ligation and start to unpair from P10 suggests that the contact between P2.1 and undocked P1-P10 might favor strand dissociation for freeing the ligated exons. Besides, the U•G wobble in P1 and the U•U mispair in P10 may also contribute to the unzipping of the ligated exons from the IGS by breaking the connectivity in base pairing between P1 and P10 helices (Fig. 3d).

### Comparisons of active sites and critical metal ions between different splicing states

**Fig. 5:**
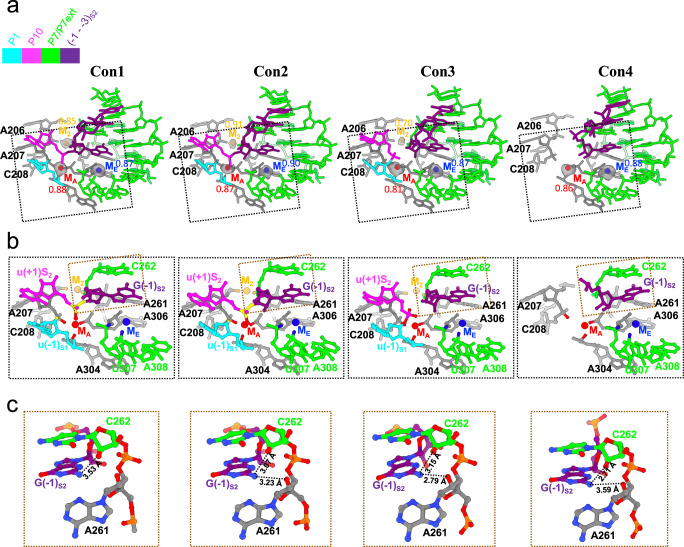
Comparison of active sites among different splicing states. **a** Overview of the active site with critical metal ions in Con1-4. The active sites of Con2-4 were aligned with reference to that of Con1. The metal ions are displayed in cryo-EM densities with the models fitted and Q-scores indicated. **b** Coordination of critical metal ions. The M_A_, M_E_, and M_2_ with their corresponding ligands are shown in red, blue, and orange, respectively. The nucleophile, scissile phosphate, leaving group, and labile bond are highlighted in yellow. **c** Interactions formed between the N2 of G(−1)_S2_ and 2′-OH groups of A261-C262 in different states.

A preorganized active site for catalysis in *Tetrahymena* ribozyme has been confirmed in previous studies^4,9^. Although the overall architecture of the active site is conserved, with root mean square deviations (RMSD) of pruned atom pairs of <0.5 Å, overlaying the atomic models of the active site from different splicing states reveals differences in the IGS/IGSext (P1-P10 helices) and J5/4 regions (Fig. 5a and Supplementary Fig. 8a–c). As described above, P1-P10 helices move out of the active site after the exon ligation (Con4 in Fig. 5a). A localized conformational shift of J5/4 was also observed in Con4 (Supplementary Fig. 8a, b). Its movement away from the active site could create a relaxed environment, possibly allowing the duplex to separate from the active site.

In addition to these significant structural alterations, some subtle rearrangements also occur in the active site, including a specific hydrogen bond and metal ions. In contrast to the hydrogen bond formed between the 2′-OH group of C262 and the exocyclic amino (N2) group of guanosine substrate in all conformations, the 2′-OH of A261 forms a hydrogen bond with the N2 of guanosine substrate selectively. This hydrogen bond is not formed in Con1 but instead forms in Con2-4 (Fig. 5b, c). These results provide a possible structural explanation for the previous finding that disruption of this interaction does not affect substrate docking but adversely affects the transesterification reaction within the ribozyme^36^. The key role of this hydrogen bond in catalysis may also be attributed to subtle conformational alterations transmitted by the base triple A261-A265-U310, which is part of the base triple sandwich in the active site.

The group I intron, as a metalloenzyme, needs a cluster of metal ions (M_A_-M_E_) to support its folding and ribozyme activity, as demonstrated by metal-ion specificity switch experiments and structural information^37–43^. M_A_ interacts with the 3′-OH oxygen at the 5′ splice site and the nonbridging oxygen of the scissile phosphate; M_B_ coordinates the 3′-OH oxygen of the guanosine substrate (ωG); M_c_ interacts with the 2′-OH oxygen of the guanosine substrate and the nonbridging oxygen of the scissile phosphate; M_D_ contacts with the residue immediately 3′ of the cleavage site. Different from the above four metal ions in or near the active site, M_E_ resides outside the active site and interacts with the nonbridging phosphate oxygens of residues U307 and A308. Based on these criteria, we search for metal ions in the cryo-EM density maps of Con1-4 and validate them using Q-scores (Fig. 5a). The potential ligands to M_A_ include the 3′-OH oxygen of u(−1)_S1_ and the nonbridging oxygen of the scissile phosphate from the exon, as well as nonbridging phosphate oxygens of C208, A304, and A306 from the intron. The metal-exon interactions consistently exist before and after exon ligation (Con1-3 in Fig. 5b and Supplementary Fig. 8d) but are lost after P1-P10 undocking (Con4 in Fig. 5b and Supplementary Fig. 8d). However, M_A_ consistently makes contact with the same nonbridging phosphate oxygens within the intron in all conformations. The M_E_ metal interactions to the nonbridging phosphate oxygens of U307 and A308 were observed in all conformations. Although these metal ions are held into place during self-splicing, they undergo subtle changes in position to coordinate with catalysis (Fig. 5b and Supplementary Fig. 8d). Since M_A_ directly contacts the atoms involved in the chemical reaction, it is closely related to the self-splicing process and interacts most strongly with the intron in Con2, priming it for chemical transformation. A similar phenomenon has been observed for M_E_ even though its interactors are not in direct contact with the docked substrate. We also observed another peripheral metal ion M_2_ that was reported to coordinate with the nonbridging phosphate oxygens of A207 and C262^9^ in Con1-3, with the strongest interaction with the intron in Con2 as well. The absence of M_2_ in Con4 can be derived from the conformational shift of J5/4, where its ligand A207 resides (Fig. 2a and Supplementary Fig. 8b). Notably, M_2_ has been observed in apo *Tetrahymena* ribozyme^9^ (Supplementary Fig. 9), indicating that M_2_ repositioning may occur before substrate binding in in vitro multi-turnover reactions. However, the functional significance of M_2_ is speculative and needs to be validated biochemically in the future. Taken together, three metal ions (M_A_, M_E_, and M_2_) were found in our structures, while there is no solid structural evidence for M_B_, M_c_, and M_D_; conformational changes occur at the intron active site during self-splicing, which likely, in turn, moves the metal ions into functional positions for catalysis. Notably, Q-scores^30^ were applied to measure the resolvability of individual residues and metal ions (Fig. 5 and Supplementary Fig. 5). Q-scores of the regions mentioned have average Q-scores above the expected levels for these resolutions (2.35-2.68 Å), allowing us to confidently analyze the subtle rearrangements at the active site (Fig. 5 and Supplementary Fig. 8). Although the metal M_c_ was modeled in our previous work^9^, it is ignored from the series of structures solved here due to its low Q-scores. One possibility for the low Q-scores of M_c_ is that phosphorothioate substitution interferes with the binding of this metal ion^44^. Besides, some base-pairing contacts around J2/2.1 differ from one structure to another, which could also be due to low Q-scores at the periphery (Supplementary Figs. 4 and 5).

## Discussion

By adding different substrates to a model ribozyme, we can mimic different steps of self-splicing. In this study, we aim to understand the structural basis for the *Tetrahymena* ribozyme catalysis and the conformational changes that accompany the second step of the self-splicing cycle using two substrates: S1 mimics cleaved 5′-exon and S2 is an analog of the 3′-splice site. Using the advantages of single-particle cryo-EM to analyze highly heterogeneous or flexible samples, we resolved six structures of holo *Tetrahymena* ribozyme. The six structures corresponding to the intermediate pre-2S state, pre-2S state, post-2S state, pre-release state, and two releasing states were achieved from a single specimen. Five out of six structures are better than 3-Å resolution with the highest one at 2.35 Å, which is the highest resolution cryo-EM structure of RNA-only to date. These resolutions enable us to determine the atomically detailed structures corresponding to different splicing intermediates and reveal the conformational changes during self-splicing.

To our knowledge, this is one of few studies of multiple RNA-only structures derived from a single specimen^45,46^, and also the structural evidence that the holo-ribozyme state can be resolved into substates. And every substate provides new information during the catalysis process: P1 helix formation and docking into the active site, followed by P7ext formation-guided 3′-exon binding and partial P10 formation in Con1 (intermediate pre-2S state); intact P10 helix formation places the 3′-splice site near the G-binding site, ready for the second transesterification reaction in Con2 (pre-2S state); exon ligation in Con3 (post-2S state); P1-P10 duplex detaches from the active site, reaches the substrate-free site in Con4 (pre-release state), and undergoes the unpairing process to release the product in Con5-6 (releasing states). Because these different conformations are obtained from the same reaction pot, they are representative of what is truly happening during one reaction process. Su et al.^9^ and Liu et al.^16^ recently reported cryo-EM structures of the *Tetrahymena* group I intron. Their highest overall resolutions are 3.06 Å and 2.98 Å (~3.0 Å and 2.85 Å for the core), respectively, which are worse than our best-resolved structure, Con2, at 2.35-Å resolution (~2.0 Å for the core). It is worth noting that they captured a single holo state at the pre-2S state (Con2 in our study) or post-2S state (Con3 in our study). Compared to these previous studies with only one holo-structure, our new continuous six structures clearly reveal a detailed structural and mechanistic view of this paradigmatic group I intron undergoing the second self-splicing process.

How the P1-P10 movement is driven to occur during the con3-con4 transition remains a mystery. Here, we offer a potential explanation: some extra density can be seen near the end of P10 in Con3 (Supplementary Fig. 7b), which may be a result of the P10 helix flexibility, favoring the duplex separation from the active site. P10 flexibility may be caused by the lack of tertiary contacts holding the duplex to the ribozyme active site after its breakage with 5′UCG_S2_, which anchors P10 in the active site via base-pairing interactions (P7ext) to the ribozyme core. Loss of the covalent connection to the duplex P7ext may promote this Con3-Con4 transition. Furthermore, in Con4, the undocked P1-P10 is stacked in the continuity of P2 after ligation and contact with P2.1, which may anchor the P1-P10 duplex to undergo the product-releasing process.

Previous research has shown that exogenous guanosine binding to the *Tetrahymena* ribozyme is much slower than the diffusion limit, and there are indications that the guanosine binding or activation may occur in two steps^13,47,48^. Based on our high-resolution structures, it is tempting to speculate that guanosine substrate binding likely also occurs in several steps in the second self-splicing reaction. The preliminary recognition of the guanosine substrate may involve the formation of P7ext and quadruple base triples, followed by the formation of P10. Following guanosine binding, subtle rearrangements of the active site occur to promote the formation of additional contacts between the intron and the substrate (like the hydrogen bond formed between the 2′-OH of A261 and the N2 of guanosine), as well as the movement of metal ions, contributing to catalysis.

Our structures combined with previous structural and biochemical data^19,49^ could support a four-step model for substrate docking and release during the second step of RNA splicing from the point of view of metal ions. Prior to the ligation reaction, the 3′-exon substrate becomes fully docked in the ribozyme (Con1-2) through tertiary contacts and metal coordination. As the reaction progresses, the ribozyme forms a stable intermediate conformation (Con3), where the exons are ligated, the metal-exon coordination remains but weakens^50^, and the tertiary interactions between 3′-exon and intron are lost. The ribozyme then adopts a relaxed docked conformation, where metal-exon coordination is lost, promoting the product duplex undocking^19,49^. However, this stable conformation for the *Azoarcus* intron was not obtained for the *Tetrahymena* ribozyme. Such variation could be attributed to sequence and research method differences. Finally, the 5′-exon loses tertiary interactions with the intron and the exon product becomes undocked, followed by duplex unpairing to release the product (Con4-6).

We now have 3D structural information for six splicing intermediates of *Tetrahymena* ribozyme following the first transesterification reaction. It is foreseeable that as the missing gaps are filled, the functions of ribozyme will be better dissected at the atomic level. In the end, one would expect to observe a molecular movie composed of a whole series of reactions and understand how the group I introns with various tertiary structures complete the same chemical transformations. As its application to structurally heterogeneous molecules continues to advance, we expect cryo-EM to become a key tool for exploring more and more biologically interesting research topics, like folding^45^ and assembly, across RNA systems.

## Methods

### RNA preparation

*Tetrahymena* L-16 ScaI ribozyme RNA sample (Supplementary Data 1) was prepared as follows. The DNA template was amplified from the pUC57-16 plasmid using the forward primer 5′-TAATACGACTCACTATAGGTTTGGAGGGAAAAGTTATCA-3′ and the reverse primer 5′-(MeA)(MeC)TCCAAAACTAATCAATATACTTT-3′. “Me” refers to the Methoxyl modification, which was introduced to ensure the integrity and accuracy of the 3′-end of the transcribed RNA sequence (Supplementary Data 1).

RNA was prepared through in vitro transcription in a reaction containing 0.315 μM DNA template, 40 mM Tris, pH 7.9, 20 mM MgCl_2_, 2 mM Spermidine, 0.01% TritonX-100, 4 mM DTT, 2 mM NTPs, 1 U/µL recombinant ribonuclease inhibitor (TaKaRa), and 7 mg/mL laboratory-purified T7 RNA polymerase. The transcription reaction was performed at 37 °C for 4 h. The RNA was purified on an 8% 19:1 acrylamide:bis, 8 M urea polyacrylamide gel. The gel was first pre-run at 260 V for 20 min to remove ions from the gel that increase conductivity. Then RNA was mixed with a loading buffer containing 95% formamide, 5 mM EDTA, 0.025% SDS, 0.02% xylene cyanol, and 0.02% bromophenol blue, and loaded on the gel. The gel was run at 300 V for 6 h and visualized briefly with a 254-nm UV lamp. Target RNA was eluted from the gel with the binding solution containing 300 mM NaOAc, pH5.2, and 1 mM EDTA by rotating at 4 °C overnight, and precipitated with the same volume of isopropanol at −80 °C overnight. After concentration and ethanol precipitation, the RNA was dissolved in RNase-free water. The RNA oligonucleotide substrates S1 (5′-FAM-cccucu or 5′-cccucu) and S2 (5′-UCG*uaacc-FAM or 5′-UCG*uaacc), in which * indicates a phosphorothioate bond, were purchased from Accurate Biotechnology (Hunan) Co., Ltd (Supplementary Data 1).

### Second-step self-splicing reaction assay

*Tetrahymena* ribozyme RNA (1 μM) in 50 mM Na-HEPES, pH 8 was denatured at 90 °C for 3 min and cooled to room temperature for 10 min. MgCl_2_ was then added to a final concentration of 10 mM and incubated at 50 °C for 30 min. The sample was cooled again to room temperature for 10 min. Afterward, substrates S1 and S2 with FAM labels (1 μM final concentration each) were added and incubated at room temperature for 20 min to form the holoenzyme complexes. The sample was then placed on ice and 5 μL of the sample was taken out at different time points: 10 min; 0.5 h; 1 h; 2 h; 3 h; 4 h; 5 h. The reaction was stopped by adding 5 μL 2X RNA denaturation loading buffer (95% formamide, 5 mM EDTA, 0.025% SDS, 0.02% xylene cyanol, and 0.02% bromophenol blue), and the sample was kept on ice. The 20% 19:1 acrylamide: bis, 8 M urea polyacrylamide gel was pre-run at 150 V for 1 h at room temperature. The samples were loaded on the gel and run at 180 V for 80 min, and imaged with the fluorescence imaging system Typhoon FLA 7000 (General Electric). This experiment was repeated independently in triplicate, and its source data are provided as a Source Data file.

### Cryo-EM sample preparation

To prepare the *Tetrahymena* ribozyme sample for cryo-EM analysis, the concentrations of ribozyme and substrate RNAs were increased. Ribozyme (~20 μM) in 50 mM Na-HEPES, pH 8 was denatured at 90 °C for 3 min and cooled to room temperature for 10 min. MgCl_2_ was then added to a final concentration of 10 mM and incubated at 50 °C for 30 min. The samples were cooled again to room temperature for 10 min. Then, substrates S1 and S2 without FAM label (50 μM final concentration each) were added and incubated at room temperature for 20 min to form the holoenzyme complexes before being placed on ice for another 5 h. Three microliters of the *Tetrahymena* ribozyme sample were applied onto glow-discharged 200-mesh R2/1 Quantifoil copper grids. The grids were blotted for 4 s and rapidly cryocooled in liquid ethane using a Vitrobot Mark IV (Thermo Fisher Scientific) at room temperature and ~100% humidity.

### Cryo-EM data collection

The grids were screened using a Talos Glacios cryo-electron microscope (Thermo Fisher Scientific) operated at 200 kV. The grids were imaged in a Titan Krios cryo-electron microscope (Thermo Fisher Scientific) operated at 300 kV at a magnification of 105,000 × (corresponding to a calibrated sampling of 0.82 Å per pixel). Micrographs were recorded by EPU software (Thermo Fisher Scientific) with a Gatan K3 Summit direct electron detector, where each image was composed of 30 individual frames with an exposure time of 3 s and a dose rate of 17.6 electrons per second per Å^2^. Finally, a total of 25,306 movie stacks were collected with a defocus range of −0.5–−2.5 μm.

### Image processing

All micrographs were motion-corrected using MotionCor2^51^ and the contrast transfer function (CTF) was determined using CTFFIND4^52^. All particles were autopicked using the NeuralNet option in EMAN2^53^ and further checked manually. The resulting number of boxed particles was 4,255,846. Then, particle coordinates were imported to Relion^54^, where three rounds of 2D classification were performed to remove 2D class averages with less resolved features. The selected 1,849,818 particles were imported to cryoSPARC^55^ for generating ab-initio maps, and two good conformations with a discernible difference in the 5′-end of the intron were derived. Then starting with the two conformations, we performed the Non-Uniform Refinement together with Local and Global CTF Refinement, yielding two maps with 2.36-Å resolution from 980,403 particles and 2.73-Å resolution from 468,004 particles. Further 3D Variability Analysis was performed to classify slightly different conformations in each of the above two classes, and totally six conformations were obtained. Final maps were achieved after another round of Non-uniform Refinement for each of the six classes, with resolutions at 2.68 Å, 2.35 Å, 2.62 Å, 2.65 Å, 2.97 Å, and 3.41 Å, respectively. To be noted, the conformational difference between Con2 and Con3 was found during model building. The cited resolutions for the final maps were estimated by the 0.143 criterion of FSC curve in cryoSPARC. (See more information in Supplementary Fig. 2 and Supplementary Table 1).

### Model building

The initial models were built using DRRAFTER^26^. The atomic model of full-length holo *Tetrahymena* ribozyme (PDB ID: 7EZ2)^9^ was first rigidly fitted into the cryo-EM densities of Con1-6. Two rounds of modeling were performed for each conformation. Then the top-scoring models of Con1-6 were subjected to model optimization with Coot^27^, such as the P9a and 5′UCG_S2_ in all conformations, the phosphorothioate bond of S2 in Con1-2, and some single-stranded linker regions. The resultant models were refined using phenix.real_space_refine with secondary structure and geometry restraints^28^. The quality of final models was evaluated by MolProbity^29^ and Q-scores^30^. Statistics of the map reconstruction and model optimization are summarized in Supplementary Table 1 and Supplementary Fig. 2. All figures were made using Chimera^56^ or ChimeraX^57^.

### Reporting summary

Further information on research design is available in the Nature Portfolio Reporting Summary linked to this article.

## Supplementary information

Supplementary Information

Description of Additional Supplementary Files

Supplementary Data 1

Supplementary Movie 1

Reporting Summary

## Data Availability

Cryo-EM structures and atomic models generated in this study have been deposited in the wwPDB OneDep System under EMD accession codes EMD-33812, EMD-33813, EMD-33814, EMD-33815, EMD-33811, EMD-33816 and PDB ID codes under accession codes 7YG9, 7YGA, 7YGB, 7YGC, 7YG8, 7YGD, respectively. The models (PDB ID: 7EZ0 and 7EZ2) used in this study are adapted from wwPDB OneDep System. The source data for Supplementary Fig. 1a are provided as a Source Data file. Source data are provided with this paper.

## Source data

Source Data
