# Supplementary material for: Snapshots of the second-step self-splicing of Tetrahymena ribozyme revealed by cryo-EM
Source: Nat Commun. 2023 Mar 16;14:1294. doi: 10.1038/s41467-023-36724-5 (PMC10020454; doi:10.1038/s41467-023-36724-5)
Supplement: Supplementary file 1 — Supplementary Information [file 41467_2023_36724_MOESM1_ESM.pdf]

Supplementary Information

Snapshots of the Second-step Self-splicing of *Tetrahymena* Ribozyme Revealed by Cryo-EM

Shanshan Li[1,*], Michael Z. Palo[2], Xiaojing Zhang[1], Grigore Pintilie[3], Kaiming Zhang[1,*]

[1]Department of Urology, The First Affiliated Hospital of USTC, MOE Key Laboratory for Cellular Dynamics, Hefei National Research Center for Interdisciplinary Sciences at the Microscale, Division of Life Sciences and Medicine, University of Science and Technology of China, Hefei 230001, China

[2]Department of Biochemistry, Stanford University, Stanford, CA 94305, USA

[3]Department of Bioengineering, Stanford University, Stanford, CA 94305, USA

[*]Corresponding author.

Emails: kmzhang@ustc.edu.cn, lishanshan@ustc.edu.cn

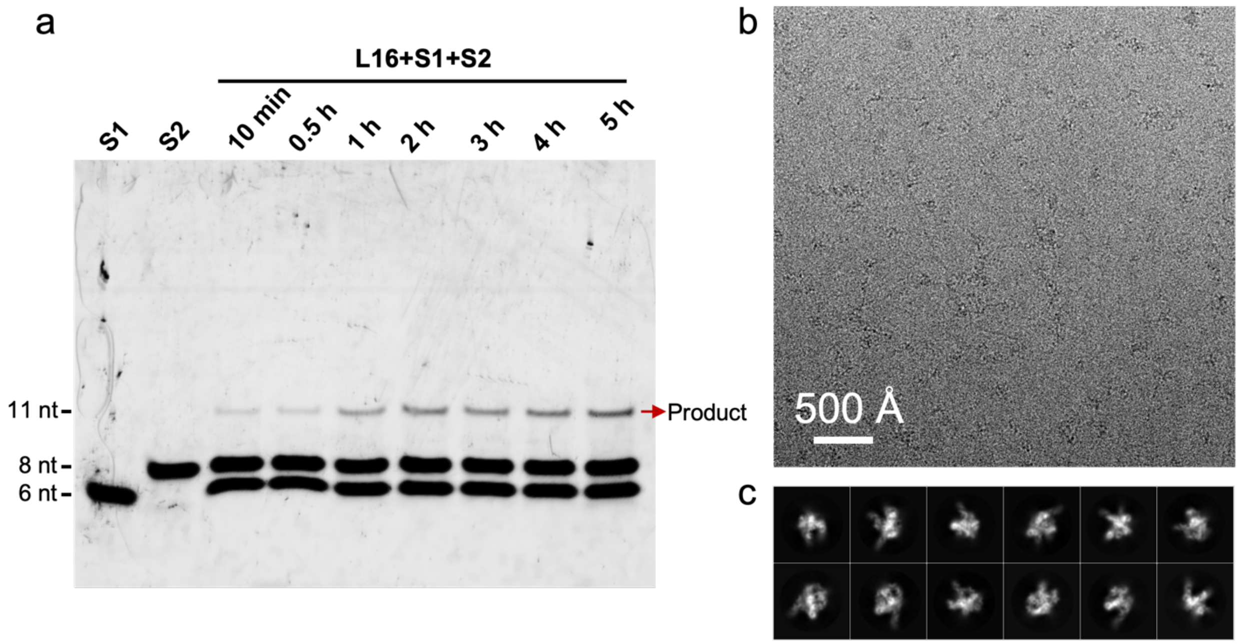

Supplementary Figure 1. Single-particle cryo-EM analysis of L16 *Tetrahymena* ribozyme. a The *in vitro* splicing assay that mimics the second-step of splicing. This experiment was repeated independently in triplicate. Source data are provided as a Source Data file. **b** Representative motion-corrected cryo-EM micrograph, selected from 24,982 micrographs. **c** Reference-free 2D class averages.

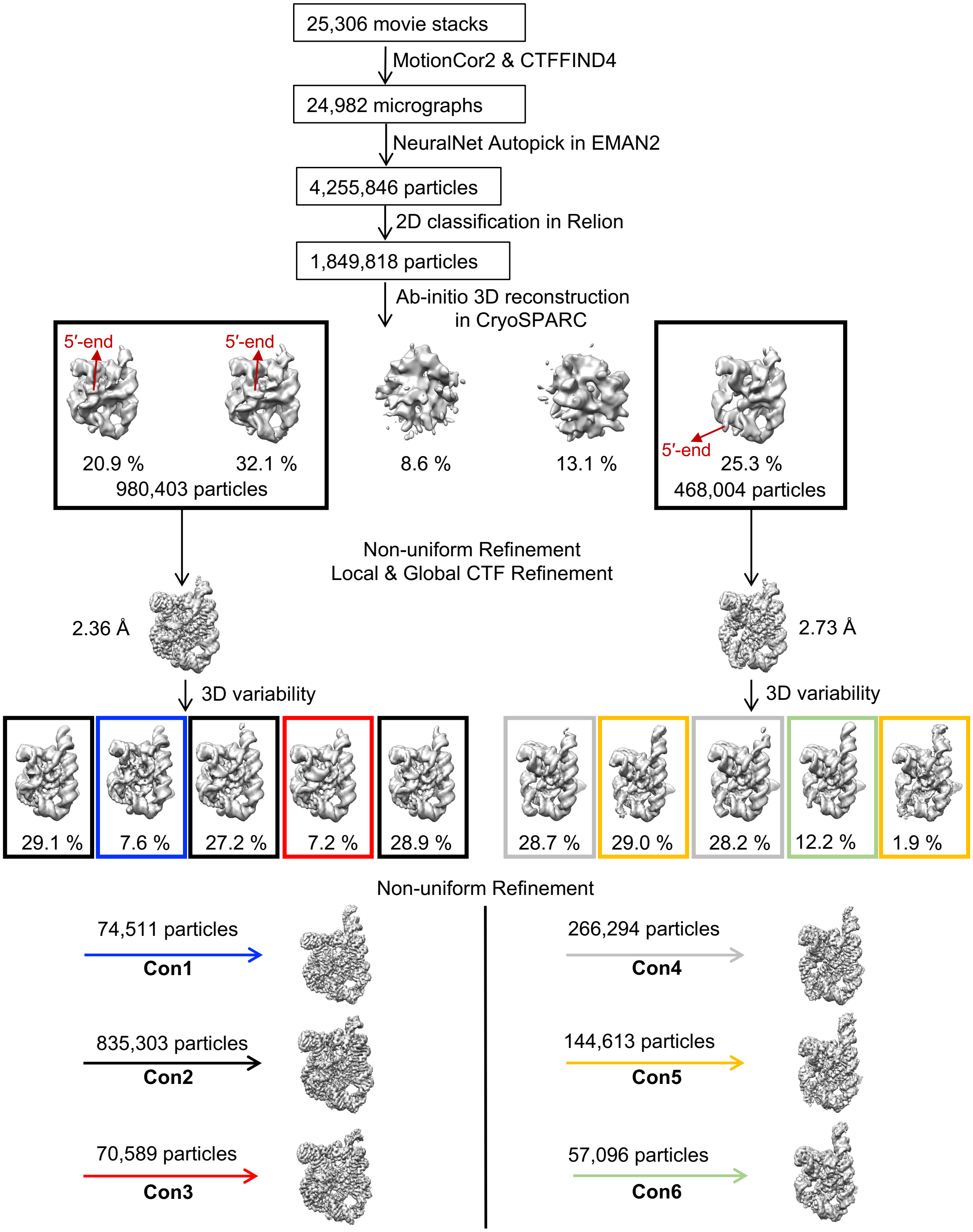

Supplementary Figure 2. Workflow of the single-particle cryo-EM data processing.

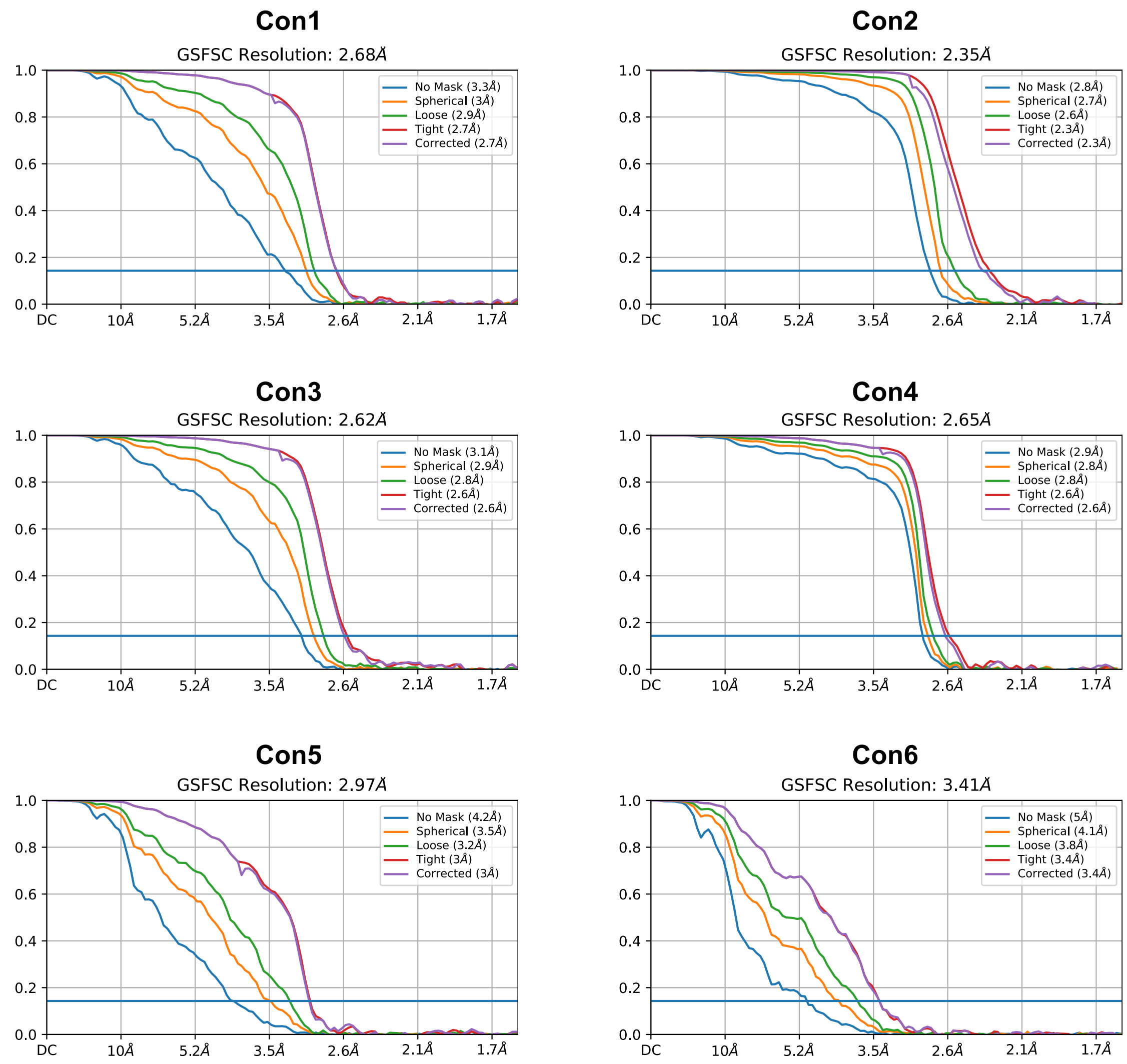

Supplementary Figure 3. Resolutions of six splicing states of *Tetrahymena* ribozyme. Gold standard FSC plots calculated in cryoSPARC.

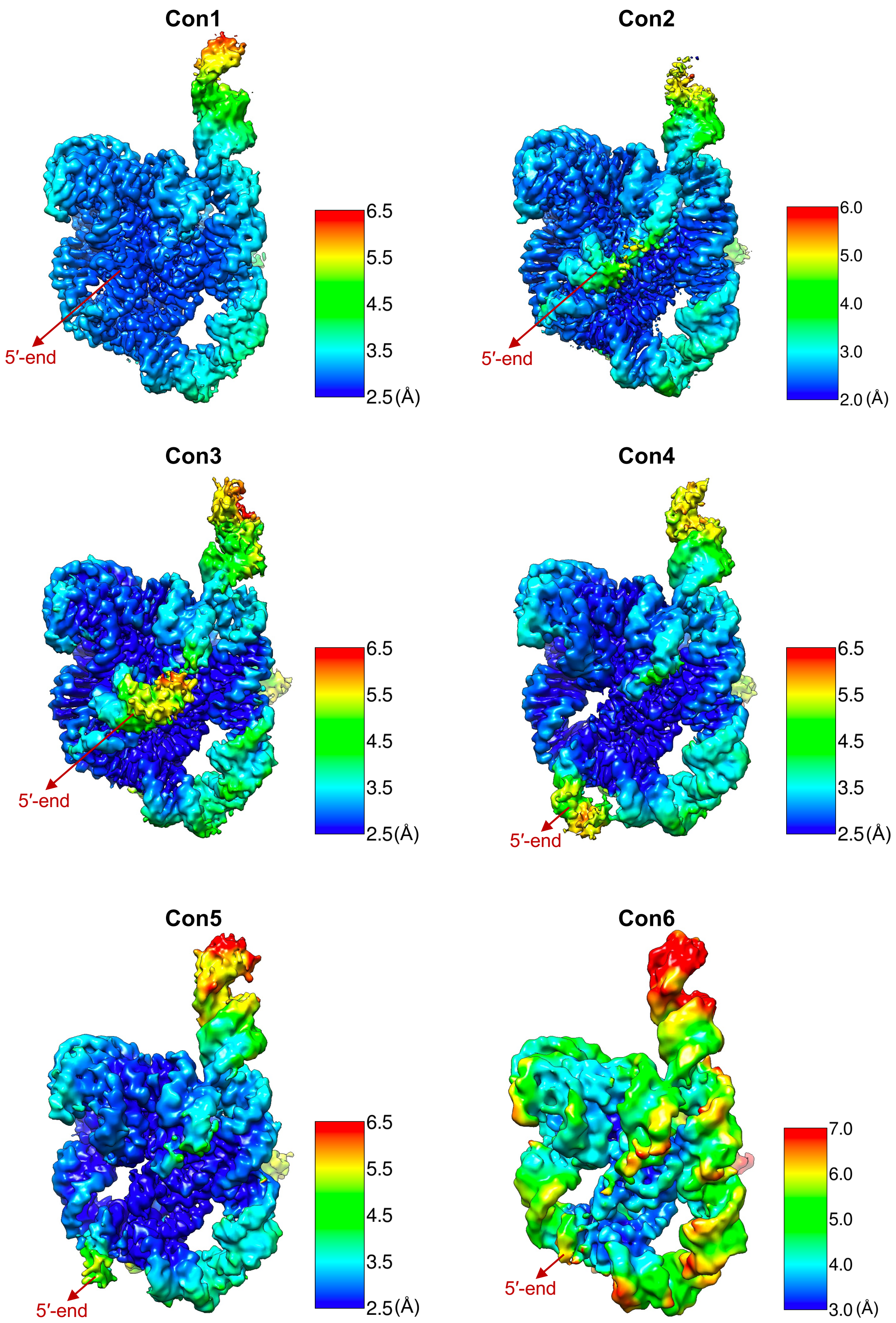

Supplementary Figure 4. Resolution maps for the final 3D reconstructions of *Tetrahymena* ribozyme.

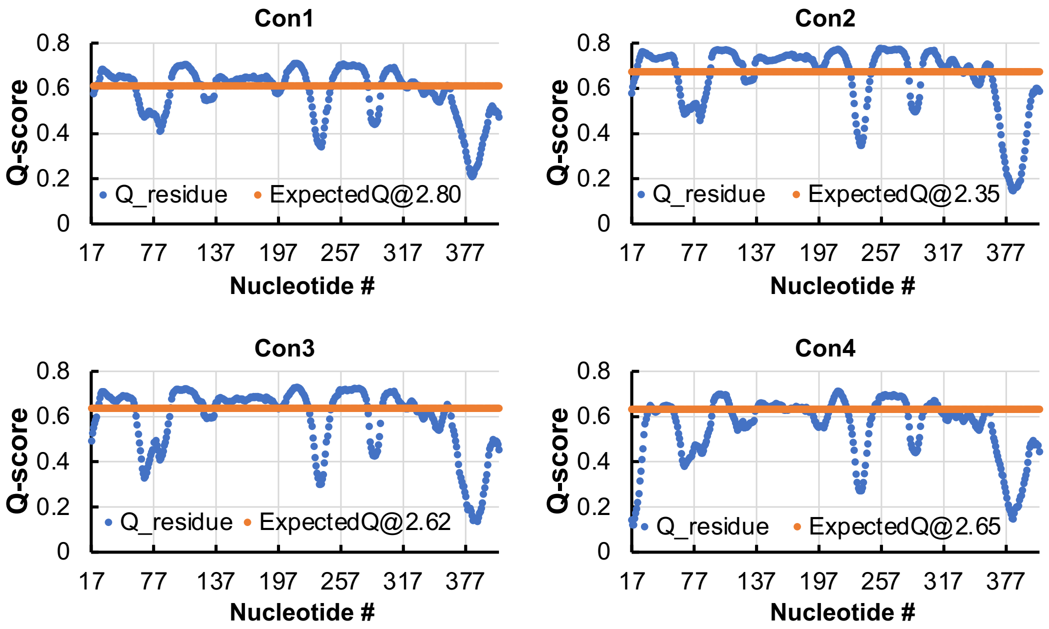

Supplementary Figure 5. Model validation using Q-scores. The orange dotted line represents the expected Q-score at respective resolution based on the correlation between Q-scores and map resolution.

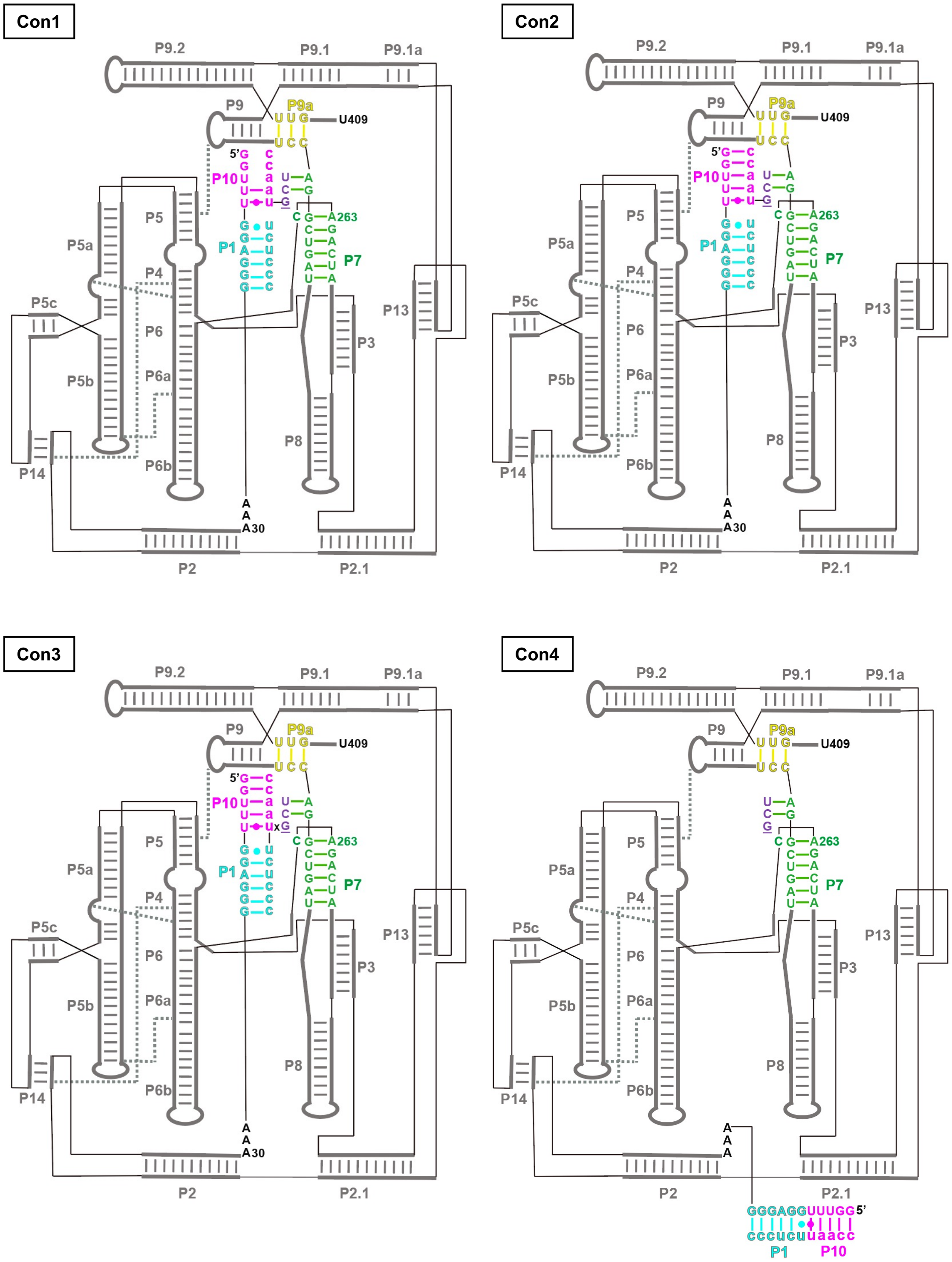

Supplementary Figure 6. Secondary structure diagrams of Con1-4. Secondary structure diagrams were adapted from RiboDraw (https://github.com/ribokit/RiboDraw) and made with Adobe Illustrator. Exon sequences are shown in lowercase and intron sequences are shown in UPPERCASE. Main tertiary contacts are shown as grey dashed lines.

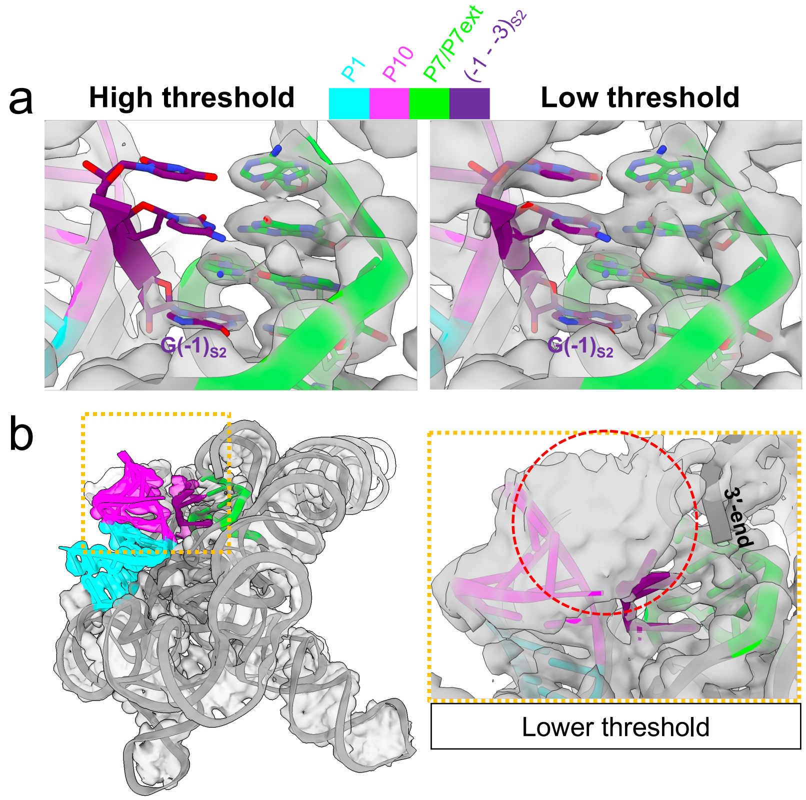

Supplementary Figure 7 related to Fig. 3. Zoom-in map and model view of the active site. a The Con3 cryo-EM map is displayed at two different thresholds to show the resolvability of 5′UCG$_{S2}$. **b** Extra cryo-EM density (red dotted circle) possibly due to P10 duplex movement in Con3.

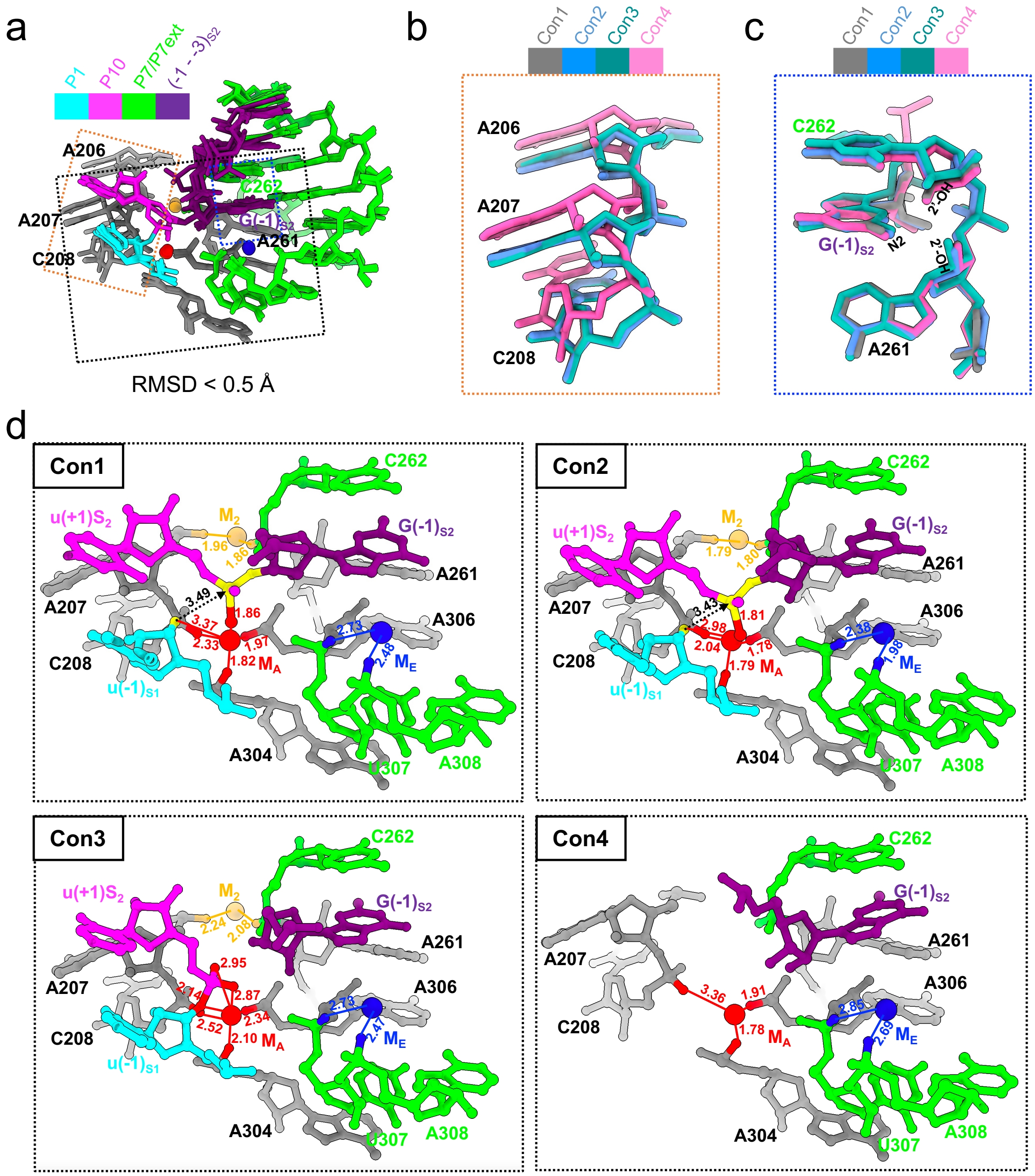

Supplementary Figure 8 related to Fig. 5. Comparison of active sites among different splicing states. **a** Superimposition of the active sites of Con1-4, with Con1 as the reference. **b** Conformational shift of A206-208 (J5/4) in Con4 compared with other conformations. **c** Spatial distributions of the 2'-OH groups of A261 and C262 relative to the N2 group of G(-1)$_{S2}$. **d** Coordination of critical metal ions. The M_A, M_E, and M_2 with their corresponding ligands are shown in red, blue, and orange, respectively. The atom distances (Å) are indicated using the same coloring scheme. The nucleophile, scissile phosphate, leaving group, and labile bond are highlighted in yellow.

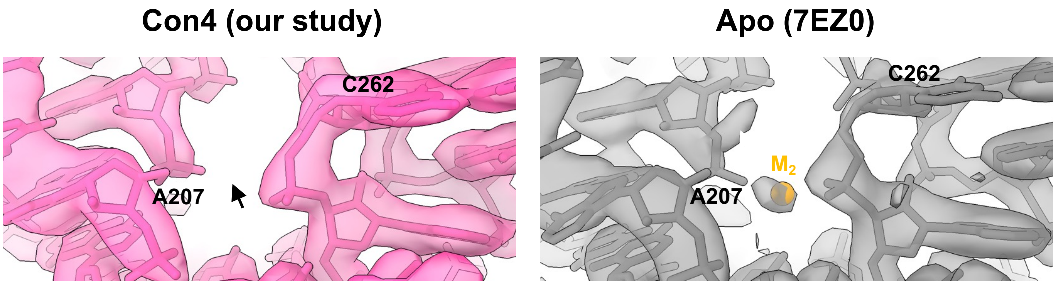

Supplementary Figure 9. Comparison of the M_2 between the *Tetrahymena* ribozyme in Con4 (our study) and apo state (7EZ0). Atomic models were fitted into cryo-EM maps (semi-transparent). The black arrowhead indicates the hypothetical position of the metal ion M_2 in Con4.

Supplementary Table 1. Cryo-EM data collection, processing, and model validation

	Holo *Tetrahymena* ribozyme					
**Data collection and processing**						
Microscope	Titan Krios G3i					
Voltage (kV)	300					
Camera	Gatan K3					
Grids Type	R2/1 Quantifoil copper grid (200 mesh)					
Sample concentration	~20 µM					
Magnification	105,000×					
C2 aperture size (µm)	70					
Objective aperture size (µm)	100					
Pixel size (Å)	0.82					
Total exposure (e-/Å^2)	52.8					
Exposure time (s)	3					
Number of frames per exposure	30					
Energy filter slit width (eV)	20					
Data collection software	EPU 2.7					
Number of exposures per hole	4					
Defocus range (µm)	-0.5 - -2.5					
Number of micrographs collected	25,306					
Number of micrographs used	24,982					
Number of initial particles	4,255,846					
Conformations	Con1	Con2	Con3	Con4	Con5	Con6
Symmetry	C1	C1	C1	C1	C1	C1
Number of final particles	74,511	835,303	70,589	266,294	144,613	57,096
Resolution (0.143 gold standard FSC, Å)	2.68	2.35	2.62	2.65	2.97	3.41
Local resolution range (Å)	2.5-6.5	2.0-6.0	2.5-6.5	2.5-6.5	2.5-6.5	3.0-7.0
**Atomic model refinement**						
Software	phenix	phenix	phenix	phenix	phenix	phenix
Clashscore, all atoms	13.88	10.8	13.12	12.48	19.24	18.65
MolProbity score	2.8	2.7	2.78	2.76	2.93	2.92
Bad bonds (%)	0	0	0	0	0	0
Bad angles (%)	0	0.01	0	0	0	0
