# Supplementary material for: Snapshots of the second-step self-splicing of Tetrahymena ribozyme revealed by cryo-EM
Source: Nat Commun. 2023 Mar 16;14:1294. doi: 10.1038/s41467-023-36724-5 (PMC10020454; doi:10.1038/s41467-023-36724-5)
Supplement: Supplementary file 2 — Description of Additional Supplementary Files [file 41467_2023_36724_MOESM2_ESM.pdf]

File Name: Supplementary Movie 1.

Description: Conformational changes of *Tetrahymena* ribozyme during the second step of self-splicing.

File Name: Supplementary Data 1.

Description: Oligonucleotides used in this study.
