# Supplementary material for: Snapshots of the second-step self-splicing of Tetrahymena ribozyme revealed by cryo-EM
Source: Nat Commun. 2023 Mar 16;14:1294. doi: 10.1038/s41467-023-36724-5 (PMC10020454; doi:10.1038/s41467-023-36724-5)
Supplement: Supplementary file 5 — Reporting Summary [file 41467_2023_36724_MOESM5_ESM.pdf]

nature portfolio

Corresponding author(s): Kaiming Zhang

Last updated by author(s): Jan 2, 2023

Reporting Summary

Nature Portfolio wishes to improve the reproducibility of the work that we publish. This form provides structure for consistency and transparency in reporting. For further information on Nature Portfolio policies, see our Editorial Policies and the Editorial Policy Checklist.

Statistics

For all statistical analyses, confirm that the following items are present in the figure legend, table legend, main text, or Methods section.

n/a	Confirmed	
☐	☒	The exact sample size (*n*) for each experimental group/condition, given as a discrete number and unit of measurement
☐	☒	A statement on whether measurements were taken from distinct samples or whether the same sample was measured repeatedly
☒	☐	The statistical test(s) used AND whether they are one- or two-sided<br>*Only common tests should be described solely by name; describe more complex techniques in the Methods section.*
☒	☐	A description of all covariates tested
☒	☐	A description of any assumptions or corrections, such as tests of normality and adjustment for multiple comparisons
☒	☐	A full description of the statistical parameters including central tendency (e.g. means) or other basic estimates (e.g. regression coefficient) AND variation (e.g. standard deviation) or associated estimates of uncertainty (e.g. confidence intervals)
☒	☐	For null hypothesis testing, the test statistic (e.g. *F*, *t*, *r*) with confidence intervals, effect sizes, degrees of freedom and *P* value noted<br>*Give P values as exact values whenever suitable.*
☒	☐	For Bayesian analysis, information on the choice of priors and Markov chain Monte Carlo settings
☒	☐	For hierarchical and complex designs, identification of the appropriate level for tests and full reporting of outcomes
☒	☐	Estimates of effect sizes (e.g. Cohen's *d*, Pearson's *r*), indicating how they were calculated

Our web collection on statistics for biologists contains articles on many of the points above.

Software and code

Policy information about availability of computer code

Data collection	EPU2.7
Data analysis	MotionCor2 1.4.0, CtfFind4.1.14, EMAN2.3, Relion3.1, DRAFFTER V1, MolProbity 4.5, Chimera 1.13.1, UCSF ChimeraX version:1.3.dev202105290025, Phenix 1.19.2-4158, Coot version:0.9.1. cryoSPARC v3.2.0

For manuscripts utilizing custom algorithms or software that are central to the research but not yet described in published literature, software must be made available to editors and reviewers. We strongly encourage code deposition in a community repository (e.g. GitHub). See the Nature Portfolio guidelines for submitting code & software for further information.

Data

Policy information about availability of data

All manuscripts must include a data availability statement. This statement should provide the following information, where applicable:

- Accession codes, unique identifiers, or web links for publicly available datasets
- A description of any restrictions on data availability
- For clinical datasets or third party data, please ensure that the statement adheres to our policy

Cryo-EM structures and atomic models generated in this study have been deposited in the wwPDB OneDep System under EMD accession codes EMD-33812, EMD-33813, EMD-33814, EMD-33815, EMD-33811, EMD-33816 and PDB ID codes under accession codes 7YG9, 7YGA, 7YGB, 7YGC,7YG8, 7YGD, respectively. The

models (PDB ID: 7EZ0 and 7EZ2) used in this study are adapted from wwPDB OneDep System. The source data for Supplementary Fig. 1a are provided as a Source Data file.

Human research participants

Policy information about studies involving human research participants and Sex and Gender in Research.

Reporting on sex and gender	N/A
Population characteristics	N/A
Recruitment	N/A
Ethics oversight	N/A

Note that full information on the approval of the study protocol must also be provided in the manuscript.

Field-specific reporting

Please select the one below that is the best fit for your research. If you are not sure, read the appropriate sections before making your selection.

☒ Life sciences ☐ Behavioural & social sciences ☐ Ecological, evolutionary & environmental sciences

For a reference copy of the document with all sections, see nature.com/documents/nr-reporting-summary-flat.pdf

Life sciences study design

All studies must disclose on these points even when the disclosure is negative.

Sample size	No statistical methods were used to predetermine sample sizes. Cryo-EM data sample size was chosen based on the sufficient number of movie stacks (totally 25,306) and particles to obtain the high resolution reconstructions.
Data exclusions	No data were excluded from the analyses.
Replication	Experiments were repeated independently at least three times. Replicate experiments were successful.
Randomization	Sample randomization is not relevant to our study, because for our structure determination, the best sample grids have to be used for data collection. The structure determination was performed following the standard procedures.
Blinding	Blinding is not relevant to cryo-EM data collection or structure analysis. Blinding is not applicable for in vitro biochemical assay.

Reporting for specific materials, systems and methods

We require information from authors about some types of materials, experimental systems and methods used in many studies. Here, indicate whether each material, system or method listed is relevant to your study. If you are not sure if a list item applies to your research, read the appropriate section before selecting a response.

Materials & experimental systems

n/a	Involved in the study
☒	☐ Antibodies
☒	☐ Eukaryotic cell lines
☒	☐ Palaeontology and archaeology
☒	☐ Animals and other organisms
☒	☐ Clinical data
☒	☐ Dual use research of concern

Methods

n/a	Involved in the study
☒	☐ ChIP-seq
☒	☐ Flow cytometry
☒	☐ MRI-based neuroimaging
